# Hypercholesteremia in Indigenous Population: The Imperative for Culturally Sensitive Interventions and Collaborative Research∗

**DOI:** 10.1016/j.jacadv.2023.100352

**Published:** 2023-05-26

**Authors:** Safi U. Khan, Khurram Nasir

**Affiliations:** Department of Cardiology, Houston Methodist DeBakey Heart & Vascular Center, Houston, Texas, USA

**Keywords:** atherosclerotic cardiovascular disease, health disparities, hypercholesterolemia, Indigenous populations, meta-analysis

**Figure:**
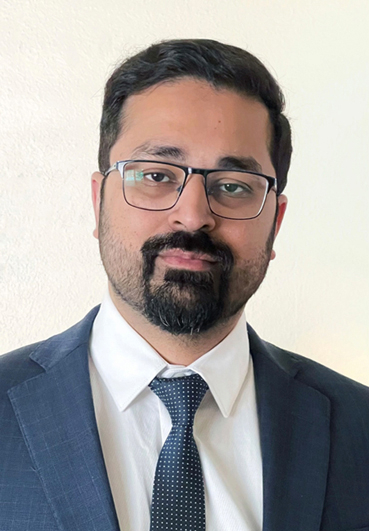

The escalating global burden of atherosclerotic cardiovascular disease (ASCVD) has prompted a growing interest in understanding the prevalence of hypercholesterolemia and its impact across diverse demographic subgroups. Familial hypercholesterolemia (FH), for example, affects approximately 1 in every 350 individuals worldwide, irrespective of race or ethnicity.^1^ Nevertheless, more than 90% of affected individuals remain undiagnosed.^1^^,^^2^ Certain subpopulations, such as French Canadians, Afrikaners, and Christian Lebanese, experience a higher frequency of FH due to the founder effect.^3^ A recent meta-analysis revealed disparities in FH prevalence among ethnic groups, with estimates ranging from 1 in 192 in African Americans to 1 in 400 in Asians.^4^ Assessing hypercholesterolemia prevalence in diverse populations is crucial for clinical research as it enhances the external validity of findings, improves the accuracy of analyses, and fosters equity in cardiovascular care.

In this issue of *JACC: Advances*, McCallum et al^5^ provided insights into the prevalence of hypercholesteremia, severe hypercholesteremia (low-density lipoprotein cholesterol [LDL-C] level ≥5.5 mmol/L [193 mg/dL]), and FH in Indigenous population. The term “Indigenous” refers to a diverse array of populations, each with unique cultural backgrounds and relationships with people and the environment.^6^ These Indigenous groups possess distinct social, cultural, economic, and political characteristics compared to the dominant societies within which they reside. The authors focused on Canada, the United States, Australia, and New Zealand for their study because these high-income countries have Indigenous minority groups that share comparable histories of colonization and forced assimilation into Western European culture.

Utilizing standard meta-analysis methodology, the authors systematically reviewed 34 studies and identified 19 studies (n = 8,662) reporting hypercholesterolemia prevalence (using various definitions) and 15 studies (n = 40,399) reporting elevated LDL-C levels based on defined cutoffs. They found no reports on the prevalence of FH in Indigenous populations within these countries. The pooled prevalence of hypercholesterolemia among Indigenous populations was estimated at 28.9%. When limiting the analysis to studies using LDL-C-based criteria, the pooled prevalence reached 25.0%, with both estimates exhibiting high levels of statistical heterogeneity. Whereas studies where a specific LDL-C cutoff of ≥3.5 mmol/L (135 mg/dL) was used, the estimated prevalence was 12.6%.

Interestingly, the review revealed that Indigenous populations in North America (Canada and the United States) had a lower hypercholesterolemia prevalence of 24.3% compared to Indigenous Australians (Aboriginal and Torres Strait Islander populations) at 40.0%. The literature search found no studies reporting on Indigenous groups in New Zealand. Lastly, a meta-regression analysis evaluated the influence of study-level characteristics (sample size, mean age, sex, year of publication, and diabetes prevalence) and demonstrated that, despite a poor predictor of variance, diabetes had a significant impact on hypercholesterolemia prevalence.

The authors appropriately recognized the limitations of their meta-analysis, such as the absence of a standardized definition for hypercholesterolemia across the studies, which likely contributed significantly to heterogeneity, and the restricted generalizability of these results to Indigenous groups ions living outside North America, Australia, and New Zealand. Additionally, this meta-analysis relied on study-level information, while patient-level data would have provided a more comprehensive understanding of the demographic and clinical characteristics of the study groups. Finally, study-level meta-regression is susceptible to ecological fallacy, which implies that the associations observed at the study level may not accurately represent the true relationships at the individual level.

## So, what can we deduce from these findings?

The study underscores the increased burden of hypercholesterolemia and ASCVD among Indigenous populations in the countries investigated. The estimated prevalence of hypercholesterolemia in the general population from these countries ranges from 28% to 33%,^7^, ^8^, ^9^ signifying that hypercholesterolemia is as prevalent among Indigenous populations as it is among non-Indigenous groups, thereby warranting its identification as a crucial public health issue. Moreover, this meta-analysis uncovers a considerable knowledge gap concerning FH and severe hypercholesterolemia within Indigenous populations. Despite being the most prevalent inherited lipid disorder, the study discovered a dearth of research on FH prevalence in these communities, with only one publication addressing the prevalence of severe hypercholesterolemia.^10^

Studies have revealed significant disparities in lipid-lowering therapy utilization rates among various demographic groups, with ethnic minority populations generally receiving fewer prescriptions and less likely to achieve cholesterol targets.^11^^,^^12^ Extending these concerns to Indigenous population, potential underdiagnosis and undertreatment of hypercholesterolemia in these patients may further contribute to elevated ASCVD rates.

## What is the path forward? Implications and recommendation

The elevated prevalence of hypercholesterolemia among Indigenous populations emphasizes the necessity of implementing targeted interventions and health care strategies to manage hypercholesteremia in these communities. Enhancing access to diagnostic tools and therapies, increasing awareness and education about hypercholesterolemia and FH, and engaging with Indigenous communities in research and health care decision-making are essential steps forward. Dedicated efforts would reduce the burden of ASCVD and improve these individuals' health outcomes and quality of life.

Additionally, the differences in the prevalence of hypercholesterolemia between Indigenous populations in North America and Australia emphasize the significance of unique cultural, social, and environmental factors contributing to these disparities. Therefore, conducting context-specific research to identify the reasons behind these differences and develop culturally appropriate interventions and community-based approaches for addressing health disparities in Indigenous populations is essential. Interventions must be developed in collaboration with Indigenous communities, respecting their unique cultural values, beliefs, and practices.

On the same note, addressing the knowledge gap in estimating FH in Indigenous populations will allow early diagnosis and treatment, ultimately reducing the risk of premature ASCVD in these communities. Moreover, the high degree of heterogeneity among studies in this review implies that there may be significant variations in hypercholesterolemia prevalence within Indigenous populations. Therefore, there is a need for extensive and standardized data collection to research potential factors driving these variations. A better understanding of these factors will facilitate tailored approaches to manage hypercholesterolemia in different Indigenous communities.

Lastly, these findings underline the need for continued investment in research, education, and public health programs to reduce health disparities in Indigenous populations. Policymakers must recognize the importance of addressing hypercholesterolemia and other chronic health conditions in these communities, allocating appropriate resources to support evidence-based interventions, and monitoring their impact over time.

In conclusion, the findings of this study have significant implications for public health, research, and policy-making. McCallum et al^5^ should be commended for their valuable contribution in shedding light on this crucial issue. By identifying the factors that contribute to these disparities and developing targeted interventions in partnership with Indigenous communities, we can work toward reducing the burden of ASCVD and improving health outcomes for these populations. By fostering collaborative efforts and investing in research, education, and public health programs, there is an opportunity to reduce health disparities and promote health equity for all.

## Funding support and author disclosures

The authors have reported that they have no relationships relevant to the contents of this paper to disclose.
